# Deep Brain Stimulation Improves Symptoms in an Individual with Alpha‐Synuclein‐Gene‐Associated Parkinson's Disease

**DOI:** 10.1002/mdc3.70057

**Published:** 2025-03-29

**Authors:** Abigail Braun, Dion Basson, Shahida Moosa, Jonathan Carr, Soraya Bardien, Riaan van Coller

**Affiliations:** ^1^ Division of Molecular Biology and Human Genetics Stellenbosch University Cape Town South Africa; ^2^ South African Medical Research Council Centre for Tuberculosis Research Stellenbosch University Cape Town South Africa; ^3^ Department of Neurology University of Pretoria Pretoria South Africa; ^4^ Medical Genetics Tygerberg Hospital Cape Town South Africa; ^5^ Division of Neurology, Department of Medicine University of Stellenbosch Cape Town South Africa; ^6^ South African Medical Research Council/Stellenbosch University, Genomics of Brain Disorders Research Unit Stellenbosch University Cape Town South Africa

**Keywords:** Parkinson's disease, *SNCA* duplication, deep brain stimulation, improvement of symptoms

Parkinson's disease (PD) is a progressive neurodegenerative disorder exhibiting a Mendelian pattern of inheritance in 5–10% of cases. Although the etiology is still largely unknown, it is widely accepted that PD is caused by aging and several genetic and environmental factors. Deep brain stimulation (DBS) is now established as a standard treatment modality for selected PD patients with levodopa‐induced motor complications and treatment‐refractory tremor. The treatment outcome of PD patients with monogenetic and genetic risk variants are of particular interest, with respect to predicting outcome from DBS, especially as genetic testing becomes more readily available. Monogenic forms of PD account for approximately 15% globally and pathogenic variants in the *SNCA* gene (including copy number variations (CNVs), that is, whole gene duplications and triplications) are rare and are found in only 0.1–0.2% of cases.^1^ Here, we report the clinical outcome of an individual with an *SNCA* gene duplication who had undergone DBS surgery.

This individual (IV‐4; Fig. S1A) is from a South African Afrikaner family (family ZA459) with autosomal dominant PD. She developed symptoms of PD in 2015 at the age of 43 with unilateral left‐sided tremor and typical gait abnormalities (Table S1). The diagnosis was confirmed clinically and with Fluorodopa (F‐DOPA) Positron Emission Tomography (PET) in 2016 (Fig. S1B–C). She responded well to levodopa therapy but developed disabling levodopa‐induced motor complications 3 years after the onset of motor symptoms, including disabling levodopa‐induced peak dose dyskinesia, on/off‐fluctuations and painful off‐phase dystonia. At the time of surgery, she was treated with 800 mg (150 mg four times daily with 200 mg at night) levodopa and 200 mg amantadine daily. Dopamine responsivity was confirmed with a documented 58% improvement in a dopamine challenge test and cognitive assessment was acceptable. Bilateral subthalamic nucleus deep brain stimulation (STN DBS) surgery was done in 2020 (Activa PC, leads 3389, Medtronic Inc., Minneapolis, Minn., USA). At the most recent clinical follow‐up in 2024 (age 52; 4 years after surgery), she still had marked benefit with a 50% reduction in levodopa equivalent daily dose (LEDD) and a 54% improvement in UPDRS‐III when compared to the pre‐surgical score (Table S2). Sustained improvement in levodopa‐induced motor complications with no off‐treatment dystonia (69% improvement in UPDRS‐IV) was reported. Also noted was an improvement post‐surgery in the UPDRS quality of life sections, 36% in UPDRS‐I and 77% in UPDRS‐II. No cognitive impairment was recorded at the last follow‐up.

Multiplex ligation‐dependent probe amplification (MLPA) analysis done in 2024 identified an *SNCA* whole gene duplication in this family, including the individual who had undergone DBS (Fig. S2).

*SNCA* gene duplications and triplications are rare with only about 60 families/probands reported to carry one of these variants, globally.^2^, ^3^ *SNCA*‐Parkinsonism presents as early‐onset PD in most cases and often with prominent non‐motor features that include dysautonomia, neuropsychiatric features and early cognitive impairment. Motor symptoms are reported to respond well to levodopa, often with early‐onset motor complications.^2^ Family ZA459 fits the description of an *SNCA* gene duplication phenotype as they exhibit an autosomal dominant pattern of inheritance and prominent non‐motor symptoms. The individual who had undergone DBS (IV‐4) and her sister (IV‐2, Fig. S1A) exhibit a young AAO of 43 years and 35 years, respectively. Limitations of our study include that video of this patient is not available and detailed neuropsychological assessments were not done.

We identified five previously reported cases of *SNCA* gene duplications and one case with an *SNCA* missense variant (c.158C > A; p.Ala53Glu) who had undergone DBS surgery (Table 1). In all cases, the target was the STN,^4^, ^5^, ^6^, ^7^, ^8^ except for a single case of juvenile‐onset (AAO of 18) with cognitive impairment, where the target was the globus pallidus internus (GPi).^9^ All of the cases with duplications improved following surgery as determined by lower motor scores, less dyskinesia and reduction in LEDD. The average (mean) reduction in LEDD in these cases (including our case) was 56 (57%). Importantly, there was no significant cognitive decline in these cases and no prominent neuropsychiatric symptoms following DBS. However, the follow‐up times of the patients varied considerably (from 1 month to 10 years).

**TABLE 1:** Reported cases of *SNCA*‐associated PD who had undergone DBS surgery

#	Reference/SCNA variant	Target	AAO/Time to surgery after diagnosis	Follow up period	UPDRS‐III^a^	Motor complications^b^	Cognitive testing^c^	Neuropsychiatric symptoms	LEDD mg/day
1	Antonini, 2012; Youn, 2022 (case 1)/Duplication	STN	41/5 years	12 months	43% improvement [28 to 16]	87% improvement [15 to 2]	20% deterioration [30 to 24]	Improved	64% reduction [1105 to 400]
2	Shimo, 2014; Youn, 2022 (case 2)/Duplication	STN	35/6 years	10 years	52% improvement [27 to 13]	+ [NR]	21% deterioration [29 to 23]	No change	35% reduction [925 to 600]
3	Perandones, 2015/Duplication	GPi	18/8 years	1 month	+ [NR]	+ [NR]	No change [NR]	No change	NR
4	Martikalnen, 2015; Youn, 2022 (case 4)/c.158C.A (p.Ala53Glu) missense variant	STN	42/5 years	3.5 years	82% Deterioration^d^ [10 to 56]^e^	96% improvement [AIMS 28 to 1]	36% deterioration [28 to 18]	Deteriorated	17% reduction [575 to 475]
5	Youn, 2022 (case 3)/Duplication	STN	37/6 years	6.5 years	35% improvement [11 to 7]^e^	+ [NR]	No change [30 to 30]	No change	68% reduction [1150 to 370]
6	Fourage, 2023/Duplication	STN	53/7 years	12 months	74% improvement [72 to 19]	100% improvement [14 to 0]	No change [NR]	No change	100% reduction [525 to 0]
7	The present study/Duplication	STN	43/4 years	4 years	54% improvement [48 to 22]	69% improvement [13 to 4]	3% deterioration [28 to 27]	No change	50% reduction [1000 to 500]
	Average (median) calculated on available data		51.6 (52)/5.6 (6) years	4.3 (3.8) years	51.6% (52%) improvement	88% (91.5%) improvement	20% (20.5%) deterioration		56% (57%) reduction

*Note*: Published or recorded scores pre‐ and post‐DBS surgery reported in square brackets.

Abbreviations: +, positive effect; −, negative effect; AAO, age‐at‐onset; AIMS, Abnormal Involuntary Movement Scale; GPi, Globus Pallidus Internus; LEDD, levodopa equivalent daily dose; MMSE, Mini Mental State Examination; MoCA, Montreal Cognitive Assessment; NR, not reported; STN, Subthalamic Nucleus; UPDRS, United Parkinson's Disease Rating Scale.

^a^ UPDRS‐III was recorded in medication‐off prior to surgery and medication‐off and stimulation‐on post‐surgery apart from cases 4 and 5 where the data in both medication‐on states were reported.

^b^ UPDRS‐IV was reported in cases 1, 6, 7 and AIMS in case 4.

^c^ Cognitive testing was reported with the MMSE in cases 1, 2, 4, 5 and with MoCA in case 7.

^d^ Case developed disabling axial symptoms with follow‐up; dyskinesia remained improved; not included in average/median scores.

^e^ UPDRS‐III reported in medication‐on state before and after surgery.

Currently, there is a global interest in the surgical outcomes of patients with monogenic PD and whether these cases have different outcomes with later follow‐up, possibly indicating different pathophysiological patterns. Notably, *GBA1* Parkinsonism pathogenic variant carriers might have more rapid cognitive decline after STN DBS, making an argument for pre‐surgical testing and consideration of other possible brain targets.^10^ However, similarly to the *SNCA* STN DBS reported cases, our case had sustained motor improvement 4 years after surgery with a significant reduction in LEDD and no post‐surgery cognitive impairment.

From this small number of cases, we postulate that individuals with *SNCA* duplications and early‐onset levodopa‐induced motor complications, without cognitive impairment, may have similar outcomes after STN DBS surgery to non‐genetic cases. Confirmation of surgical outcomes with a larger case series is required to confirm this hypothesis.

## Author Roles

(1) Research project: A. Conception, B. Organization, C. Execution. (2) Statistical Analysis: A. Design, B. Execution, C. Review and Critique. (3) Manuscript Preparation: A. Writing of the First Draft, B. Review and Critique.

A.B.: 1B, 1C, 3A, 3B.

D.B.: 1C, 3B.

S.M.: 1B, 3B.

J.C.: 1A, 3B.

S.B.: 1A, 1B, 3A, 3B.

R.v.C: 1A, 1B, 1C, 3A, 3B.

## Disclosures

**Ethical Compliance Statement:** This study was granted ethics approval from the Health Research Ethics Committee (HREC) of Stellenbosch University, South Africa (protocol number 2002C/059). All participants provided informed written consent. We confirm that we have read the Journal's position on issues involved in ethical publication and affirm that this work is consistent with those guidelines.

**Funding Sources and Conflicts of Interest:** This work is based on the research supported in part by the National Research Foundation of South Africa (grant number 129429), and the South African Medical Research Council (Self‐Initiated Research Grant). We also acknowledge the support of the Centre for Tuberculosis Research (CTR) of the South African Medical Research Council. The authors declare that there are no conflicts of interest relevant to this work.

**Financial Disclosures for Previous 12 Months:** RvC receives consultation fees from Medtronic Africa.

## Supporting information


**Figure S1.** (A) Pedigree of family ZA459. The affected individuals are the individual who had undergone DBS (IV‐4), her sister (IV‐2) and their mother (III‐2). All individuals with sample IDs were included in the genetic analysis. (B, C) F‐DOPA PET images of individual IV‐4 show significant asymmetrical reduction in dopamine receptor binding on the (B) coronal and (C) axial images. The right side being more affected corresponds to the clinical picture of left‐sided onset and dominant Parkinsonism.
**TABLE S1.** Demographic and phenotypic information for family ZA459.
**TABLE S2.** Pre‐ and post‐deep brain surgical outcomes in the family member (individual IV‐4) 4 years after surgery.
**Figure S2.** MLPA ratio charts. (A–C) Ratio charts for the three affected family members displaying the SNCA gene duplication. The ratio charts depict a ratio of 1.5 (shown in black boxes), signifying the presence of a duplication.

## Data Availability

The data that support the findings of this study are available from the corresponding author upon reasonable request.
