# Supplementary material for: Deep Brain Stimulation Improves Symptoms in an Individual with Alpha‐Synuclein‐Gene‐Associated Parkinson's Disease
Source: Mov Disord Clin Pract. 2025 Mar 29;12(8):1200–3. doi: 10.1002/mdc3.70057 (PMC12371441; doi:10.1002/mdc3.70057)
Supplement: Supplementary file 1 — Figure S1. (A) Pedigree of family ZA459. The affected individuals are the individual who had undergone DBS (IV‐4), her sister (IV‐2) and their mother (III‐2). All individuals with sample IDs were included in the genetic analysis. (B, C) F‐DOPA PET images of individual IV‐4 show significant asymmetrical reduction in dopamine receptor binding on the (B) coronal and (C) axial images. The right side being more affected corresponds to the clinical picture of left‐sided onset and dominant Parkinsonism. TABLE S1. Demographic and phenotypic information for family ZA459. TABLE S2. Pre‐ and post‐deep brain surgical outcomes in the family member (individual IV‐4) 4 years after surgery. Figure S2. MLPA ratio charts. (A–C) Ratio charts for the three affected family members displaying the SNCA gene duplication. The ratio charts depict a ratio of 1.5 (shown in black boxes), signifying the presence of a duplication. [file MDC3-12-1200-s001.docx]

## Supplementary Material

Title:  **Deep brain stimulation improves symptoms in an individual with alpha-synuclein-gene-associated Parkinson’s disease**

**(A)**


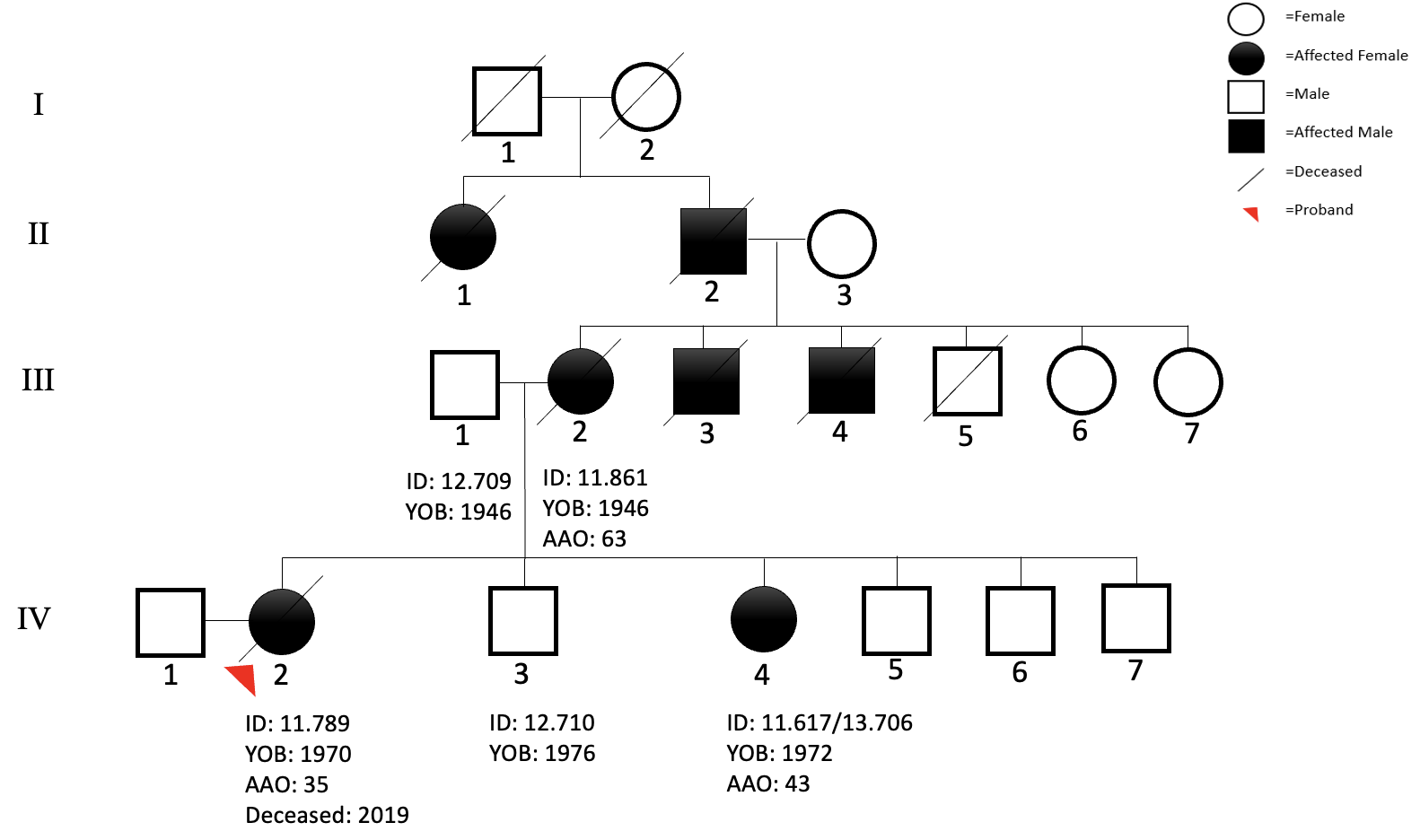


**Had undergone DBS**


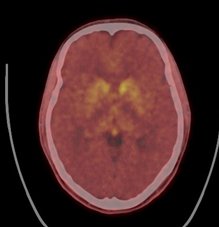

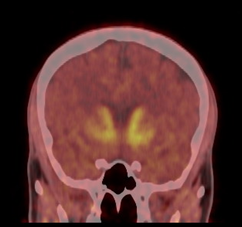


**(C)**

(C)

**(B)**

**Supplementary Figure S1**. (A) Pedigree of family ZA459. The affected individuals are the individual who had undergone DBS (IV-4), her sister (IV-2) and their mother (III-2). All individuals with sample IDs were included in the genetic analysis. (B-C) F-DOPA PET images of individual IV-4 show significant asymmetrical reduction in dopamine receptor binding on the (B) coronal and (C) axial images. The right side being more affected corresponds to the clinical picture of left-sided onset and dominant Parkinsonism.

Abbreviations: ID, sample identification; YOB, year of birth; AAO, age at onset.

**Supplementary Table S1:** Demographic and phenotypic information for family ZA459.

|  | Affected | | | Unaffected | |
| --- | --- | --- | --- | --- | --- |
| Individual | IV-2 *  (proband) | IV-4 | III-2 * | III-1 | IV-3 |
| Lab ID | 11.789 | 11.617/13.706 | 11.861 | 12.709 | 12.710 |
| Year of Birth | 1970 | 1972 | 1946 | 1946 | 1976 |
| Sex | Female | Female | Female | Male | Male |
| Age at Death | 49 | N/A | 70 | N/A | N/A |
| Deep Brain Stimulation | No | Subthalamic DBS done 20 Nov 2020 | No |  |  |
| Date of Last Clinical Evaluation | 2013 | 2024 | 2013 | N/A | N/A |
| Age at Onset (years) | 35 | 43 | 63 | N/A | N/A |
| Resting Tremor | Yes | Yes | Unknown |  |  |
| Postural Tremor | Unknown | No | Yes |  |  |
| Action Tremor | Unknown | No | Unknown |  |  |
| Slow Movements | Unknown | Yes | Unknown |  |  |
| Rigidity/Stiffness | Unknown | Yes | Unknown |  |  |
| Gait Difficulty | Unknown | Yes | Unknown |  |  |
| Gait Freezing | Unknown | No | Unknown |  |  |
| Postural Instability/Falls | Unknown | No | Unknown |  |  |
| Dystonia | Yes | Yes | Yes |  |  |
| Clear, Favourable Response to Dopaminergic Medication | Unknown | Yes | Unknown |  |  |
| Motor Fluctuations (On/Off Periods) | Unknown | Yes | Unknown |  |  |
| Levodopa-induced Dyskinesias | Unknown | Yes | Unknown |  |  |
| Sleep Benefit | Unknown | No | Unknown |  |  |
| Upper Motor Neuron Signs | Unknown | No | Unknown |  |  |
| RBD | Unknown | Yes | Unknown |  |  |
| Insomnia | Unknown | Yes | Unknown |  |  |
| Excessive Daytime Sleepiness | Unknown | No | Unknown |  |  |
| Depression Symptoms | Unknown | Yes | Unknown |  |  |
| Anxiety | Unknown | Yes | Yes |  |  |
| Mild Cognitive Impairment | Unknown | Yes | Unknown |  |  |
| Dementia | Unknown | No | Unknown |  |  |
| Visual Hallucinations | Unknown | No | Unknown |  |  |
| Impulse Control Disorders | Unknown | No | Unknown |  |  |
| Constipation | Yes | Yes | Yes |  |  |
| Urinary Dysfunction | Unknown | No | Unknown |  |  |
| Orthostatic Hypotension | Unknown | No | Unknown |  |  |
| Pain | Unknown | Yes | Unknown |  |  |
| Hyposmia | Unknown | No | Unknown |  |  |
| Underweight | Unknown | No | Unknown |  |  |
| Presence of Atypical Features | Yes  Leg Spasms and Speech Impairments | No | Yes  Scoliosis |  |  |
|  |  |  |  |  |  |
| Aggressive Behaviour | Unknown | No | Unknown |  |  |
| Childhood Symptoms | Unknown | None | Unknown |  |  |
| Initial Symptoms | Unknown | Unilateral left hand tremor | Unknown |  |  |
| Olfaction (Normal/Impaired) | Unknown | Normal | Unknown |  |  |
| Excessive Sweating | Unknown | No | Unknown |  |  |
| Total Daily Dose of Levodopa (mg) | Unknown | 500mg | Unknown |  |  |
| Blood Pressure (Presence/Absence of Orthostatic Drop) | Unknown | Normal | Unknown |  |  |
| Reflexes (Normal/Hyperreflexia) | Unknown | Normal | Unknown |  |  |
| Brain CT Scan (Done/Not Done & Findings) | Unknown | CT - F-DOPA PET - confirmed diagnosis of PD 26 Jun 2022; MRI brain normal 25 May 2020 | Unknown |  |  |
| UPDRS (Unified Parkinson Disease Rating Scale) part III | Unknown | 22 | Unknown |  |  |
| MOCA (Montreal Cognitive Assessment) | Unknown | 24/30 | Unknown |  |  |

* Clinical records of 11.789 and 11.861 were destroyed after they died so no clinical information is available

**Supplementary Table S2:** Pre- and post-deep brain surgical outcomes in the family member (individual IV-4) four years after surgery.

| **Scale** | **Pre-STN DBS surgery (2020)** | **Post-STN DBS surgery (2024)** | **Percentage change** |
| --- | --- | --- | --- |
| UPDRS -I | 11 | 7 | 36% |
| UPDRS -II | 13 | 3 | 77% |
| UPDRS -III (levodopa-off) | 48 | 22 | 54% |
| UPDRS -IV | 13 | 4 | 69% |
| Levodopa equivalent daily dose (LEDD) | 1000 | 500 | 50% |
| MoCA | 28 | 27 | 3% |

STN DBS, subthalamic nucleus deep brain stimulation; UPDRS, Unified Parkinson's Disease Rating Scale

**(A)**


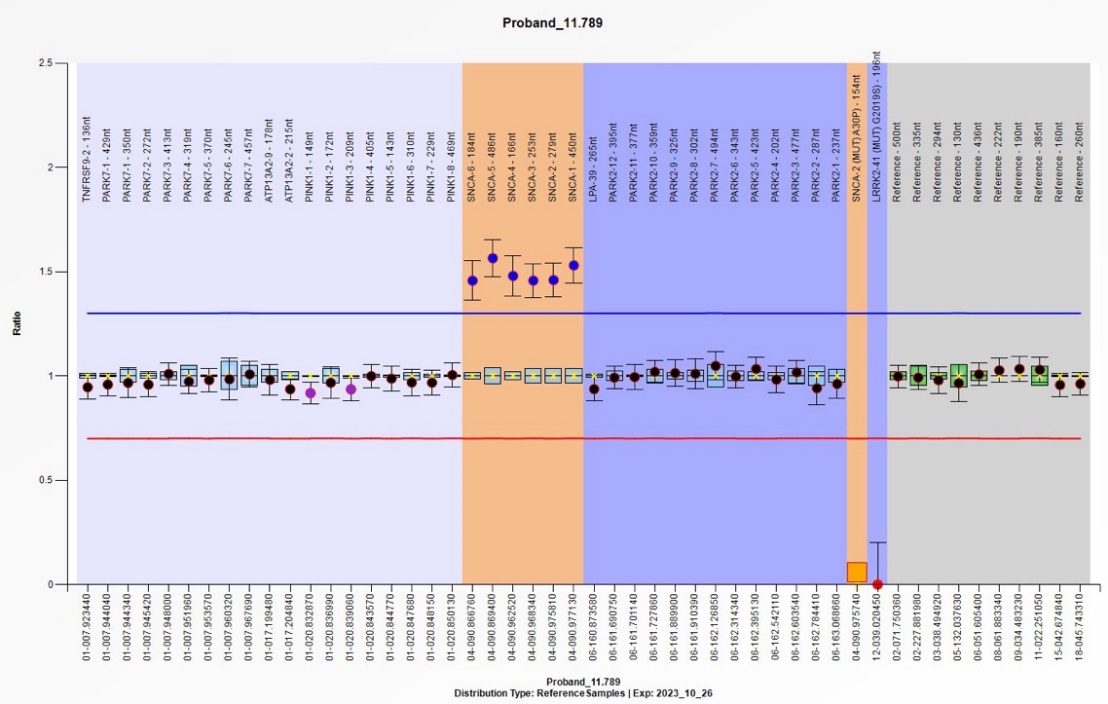


IV-2 (ID 11.789)


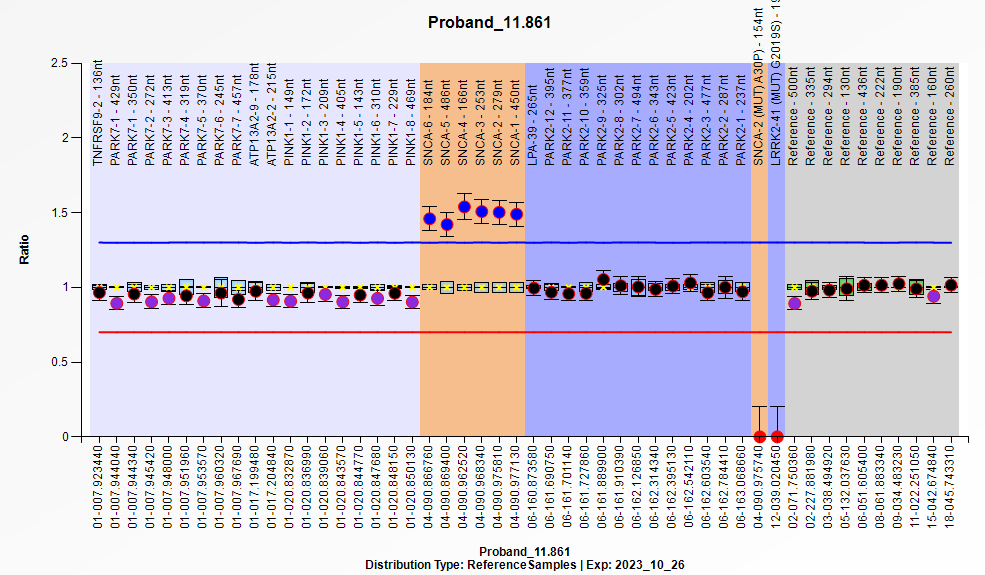


III-2 (ID 11.861)

**(B)**

IV-4 (ID 13.706)

**(C)**


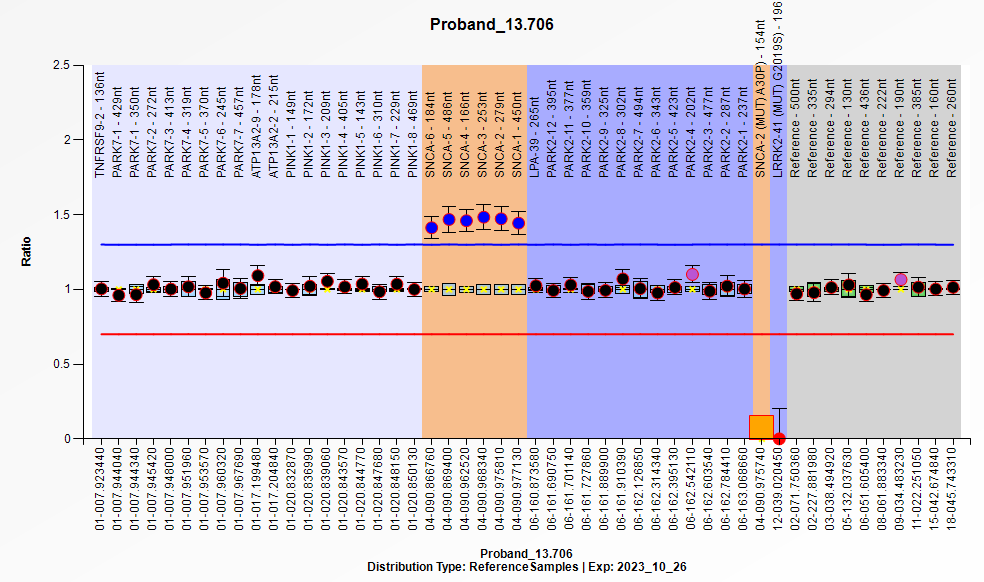


**Supplementary Figure S2:** MLPA ratio charts. (A-C) Ratio charts for the three affected family members displaying the *SNCA* gene duplication. The ratio charts depict a ratio of 1.5 (shown in black boxes), signifying the presence of a duplication.
